# Investigating the effects of climatic variables and reservoir on the incidence of hemorrhagic fever with renal syndrome in Huludao City, China: a 17-year data analysis based on structure equation model

**DOI:** 10.1186/1471-2334-9-109

**Published:** 2009-07-08

**Authors:** Peng Guan, Desheng Huang, Miao He, Tiefeng Shen, Junqiao Guo, Baosen Zhou

**Affiliations:** 1 Department of Epidemiology, School of Public Health, China Medical University, Shenyang 110001, PR China; 2 Department of Mathematics, College of Basic Medical Sciences, China Medical University, Shenyang 110001, PR China; 3 Information Center, the First Affiliated Hospital, China Medical University, Shenyang 110001, PR China; 4 Division of Infectious Diseases Control, Huludao Municipal Center for Disease Control and Prevention, Huludao 125000, PR China; 5 Liaoning Provincial Center for Disease Control and Prevention, Shenyang 110005, PR China

## Abstract

**Background:**

HFRS is a serious public health problem in China and the study on HFRS is important in China for its large population. The present study aimed to explore the impact of climatic variables and reservoir on the incidence of HFRS in Huludao City, an epidemic focus of the disease in northeastern China.

**Methods:**

Structure Equation Model (SEM), a statistical technique for testing and estimating causal relationships, was conducted based on climatic variables, virus-carrying index among rodents, and incidence of HFRS in the city during the period 1990 to 2006. The linear structural relationships (LISREL) software (Scientific Software International, Lincolnwood, IL) was used to fit SEMs.

**Results:**

Temperature, precipitation, relative humidity and virus-carrying index among rodents have shown positive correlations with the monthly incidence of HFRS, while air pressure had a negative correlation with the incidence. The best-fit SEM model fitted well with the data-based correlation matrix, P value was more than 0.56, root mean square error of approximation (RMSEA) equaled to 0, goodness-of-fit index (GFI) was more than 0.99.

**Conclusion:**

Climate and reservoirs have affected the incidence of HFRS in Huludao City, located in northeastern China. Climate affects HFRS incidence mainly through the effect on reservoir in the study area. HFRS prevention and control should give more consideration to rodent control and climate variations.

## Background

Hemorrhagic fever with renal syndrome (HFRS) is a zoonosis caused by Hantaan or Hantaan-related virus, with characteristics of fever, hemorrhage, kidney damage and hypotension. In China, HFRS was recognized in northeastern China in 1931[1]. There is a concern recently for an outbreak in some countries [2-4]. China is the most severe endemic country, with 90% of the total HFRS worldwide cases reported[1]. Although vaccination, rodent control, environment management and other related measures have been implemented, HFRS remains a serious public health problem in mainland China with 20,000–50,000 human cases annually reported[5]. Liaoning Province is one of the most serious affected areas with the most cases in mainland China and the highest incidence during the years 2004 and 2005[6], and an outbreak of HFRS was also reported in Liaoning in 2006[7]. There are two kinds of rodent-borne virus epidemic of HFRS in Liaoning province: Hantaan virus transmitted by the striped field mouse (*Apodemus agrarius*) and Seoul virus transmitted by the brown rat (*Rattus norvegicus*). Although the incidence of HFRS is stable and has a descending trend at the national level in China, the scope of epidemic focus of the Seoul virus is still expanding. Huludao city was selected as the study area because Huludao city is the traditional HFRS epidemic focus of the Seoul virus. It was recognized as an epidemic focus of HFRS in 1984 and has since served as a national HFRS surveillance site[8,9].

It has been accepted that climate plays a role in the transmission of many infectious diseases including HFRS [10-14]. Several studies have explored the association between HFRS and climate variation [15-19]. The climatic variables, such as temperature, not only affect the rate of replication of virus, but also have an impact on the environmental reservoirs-rodents [20-22]. Thus, the climatic variables are expected to influence the incidence of HFRS through rodents. Because the variation of HFRS incidence are linked inherently to the climate and reservoir factors, to what degree the relationship between climatic variables, reservoir information and HFRS is a focus of interest in the present study.

Correlation and regression analysis are common methods for existing studies on the transmission of HFRS. The limitations of these methods are that some variables, like climate and reservoir, can't be described clearly and the interaction between them could not be explored. Structural equation model (SEM)[23,24] is a statistical technique for testing and estimating causal relationships using a combination of statistical data and qualitative causal assumptions. SEMs were originally developed in the early 1970s in the field of social science of fit models with variables that cannot be measured or observed. A key feature of the SEM approach is that it allows one to compare candidate models. SEM grows out of and serves purposes similar to multiple regression. It is a combination of multiple regression and factor analysis. Recently, SEM has become a particularly attractive data-analytic option because of the development of several new types of models and software capabilities that are particularly well suited to the research interests of clinical scientists and public health managers[25,26].

In the present study, SEM was adopted to analyze the influence of climate, reservoir on the incidence of HFRS and describe the effect in quantity. It was expected that HFRS forecasts or control decision-making could be based on the relationships between HFRS and correlated climatic and reservoir factors in both surveyed and similar unsurveyed areas.

## Methods

### Study area and data collection

**Figure 1:**
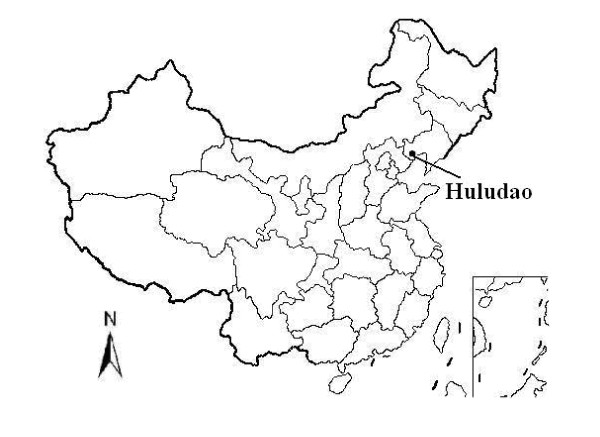
**Location of study area (Huludao City) in northeastern China**.

Huludao is a coastal city (40°N and 120°E, Figure 1) of Liaoning Province with a population of approximately 2.78 million in 2007 and a temperate climate. In Huludao, average temperature is about 8.1 to 9.2°C, with the average maximum temperature between 14.3 to 15.1°C, and the average minimum temperature between 2.3 to 4.0°C. Its annual rainfall is about 560 to 630 mm average and annual precipitation mainly concentrated in July and August. Four seasons are spring, March–May; summer, June–August; autumn, September–November; winter, December–February. Demographic information for Huludao City was collected from local government report.

Liaoning Province was the most seriously affected area by HFRS during 2004 to 2005[6], and Huludao has been recognized as an epidemic focus of HFRS since 1984. As a national HFRS surveillance site, good records about HFRS incidence and other related information have been kept in Huludao city. HFRS is a legally mandated notifiable disease in China, the Law on the Prevention and Control of Infectious Diseases[27] requires health-care staff to report any of the 37 infectious diseases, including HFRS, to the Center for Disease Control and Prevention (CDC) through the National Noticeable Infectious Disease Reporting system (NIDR). The data of monthly incidence of HFRS in Huludao from 1990 to 2006 was obtained from Huludao Municipal CDC. Huludao became a city in June 1989, before then it was a county-level city. And from 2005, vaccination against HFRS began on a large-scale in Huludao with about 100,000 people annually vaccinated. The inactivated vaccine was against both Hantaan and Seoul virus. Thus, only the data from 1990 to 2006 was collected. HFRS cases (from urban and rural areas of Huludao City) were first diagnosed based on clinical symptoms, then blood samples were collected for serologic identification which was performed at the laboratory of Liaoning Provincial CDC to confirm the clinical diagnosis. Serological identification was based on indirect fluorescent antibody test (IFAT) to study both Hantaan and Seoul virus. Detailed procedures can be found in published articles[28,29]. The patterns of HFRS notification were consistent across the study period, under the regular guidance of Liaoning Provincial CDC. Meteorological data were retrieved from the China Bureau of Meteorology. Meteorological data consisted of the corresponding monthly air pressure, temperature, precipitation and relative humidity.

Information on rodent samples (the density of rodents and the rate of virus-carrying rodents) were obtained from Huludao Municipal CDC. We used the "Virus-carrying index" to describe the combined effect of rodent density and virus-carrying rate[1]. Virus-carrying index is one way to attempt summarizing infection situation among rodents into a single numeric value. Virus-carrying index = , where density of rodents =  and virus-carrying rate among rodents = . So, virus-carrying index = . Here, the part that under the radical sign can be transformed into a fraction whose denominator is a constant, i.e. the situation that the number of the placed traps is same. The number of rodents that catch virus is very small compared with the number of traps, so it follows Poisson distribution, which is derived as a limiting case of the binomial distribution. When a variable is Poisson distributed, its square root is approximately normally distributed and easily to be compared. The other reason to select square root transformation is that both the density of rodents and virus-carrying rate among rodents are percentages, the square root of their product can maintain the consistency of unit. Based on the surveillance results from all the national surveillance spots of HFRS in China Mainland, Chen et al indicated that compared with density of rodents and virus-carrying rate among rodents, virus-carrying index one month prior was more suitable for forecasting HFRS incidence in quantity[30]. This CDC conducts a density-of-rodents survey in the residential areas and fields where the rodents may haunt four times annually (according to the China National Surveillance Plan for HFRS control). For each survey four fields are chosen in the east, west, south and north of the City. At least 300 traps are placed at each trapping site each night, and the survey is conducted over three consecutive nights: one trap every 5 meters in each row with 50 meters between rows. The number of rodents captured divided by the number of traps placed at a certain trapping site is that season's density of rodents in that field. The average of a season's density of rodents in the four different fields represents the season's density of rodents for the city[16]. In the fields, the species of rodent was identified, killed with ether, and their sera and lungs were collected and stored in a portable liquid-nitrogen freezer. Virus isolation was then performed in laboratory. Direct immunofluorescence assay (IFA) and hemagglutination inhibition test were used to identify and characterize the virus in the rodents. Details can be found in previously published articles [31-33]. The number of rodents that catch virus divided by the number of rodents captured is the season's virus-carrying rate among rodents in that field. *Rattus norvegicus *is the dominant species of HFRS rodents reservoir(Seoul virus) and also the most prevalent rodent in the surveyed area in Huludao city, accounts for more than 80% of different rodents[34]. Over the study period, no missing data was found in both the meteorological database and the disease surveillance database.

### Data analysis

#### Correlation analysis

The relationships between monthly mean meteorological factors, reservoir related variable and the monthly incidence of HFRS were examined. Pearson's correlation was performed to quantify the relationships between climatic variables, reservoir data and the monthly incidence of HFRS with a lag of zero to three months. Pearson's correlation analysis was conducted by using the Statistical Product and Service Solutions (SPSS 12.0 for windows, SPSS Inc., Chicago, IL, USA).

#### Structure equation model construction

Logarithmic transformation of HFRS incidence was performed to normalize its distribution and SEM was used to assess the independent effects of climatic and reservoir variables on the incidence of HFRS over the period 1990 to 2006 in Huludao City. The LISREL (linear structural relationships) software (Scientific Software International, Lincolnwood, IL, USA) was used to fit SEMs, and a detailed description of this software can be found in Jöreskog and Sörbom[35].

SEM for incidence of HFRS with two exogenous latent variables, i.e. climate and reservoir, was constructed. Goodness of fit was tested to determine whether the model being tested should be accepted or rejected. LISREL provided 15 different goodness-of-fit measures, the choice of which was a matter of dispute among methodologists[36]. Goodness of fit in the present study was measured on the basis of chi-square χ^2^, Goodness-of-Fit Index (GFI), Comparative Fit Index (CFI), Normed-fit Index (NFI), Standardized Root Mean Square Residual (SRMR) and Root-Mean Square Error of Approximation (RMSEA)[37]. The χ^2 ^has traditionally been used to test the hypothesis that the relationships suggested in the model provide a plausible explanation of the data. Ideally, it should be non-significant. The Goodness-of-Fit statistic (GFI) is an alternative to the Chi-Square test and calculates the proportion of variance that is accounted for by the estimated population covariance. The SRMR is the square root of the difference between the residuals of the sample covariance matrix and the hypothesised covariance model. Normed-fit index (NFI) assesses the model by comparing the χ^2 ^value of the model to the χ^2 ^of the null model. Values for this statistic range between 0 and 1 and values greater than 0.90 indicate a good fit. Comparative fit index (CFI) is a revised form of the NFI which takes into account sample size that performs well even when sample size is small. As with the NFI, values for this statistic range between 0 and 1 with values closer to 1.0 indicating good fit[35].

#### Ethical review

The present study was reviewed by research institutional review board of China Medical University and found to be utilization of disease surveillance data and meteorological data not requiring oversight by an ethics committee.

## Results

### HFRS incidence in Huludao City, 1990 to 2006

**Figure 2:**
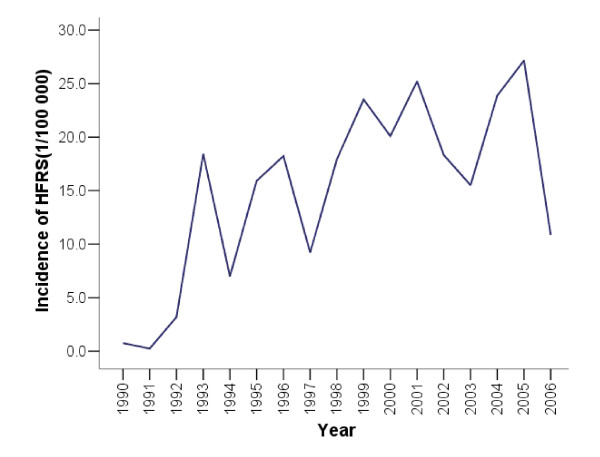
**Annual incidence of hemorrhagic fever with renal syndrome (HFRS) (Huludao City, China, 1990-2006)**.

**Figure 3:**
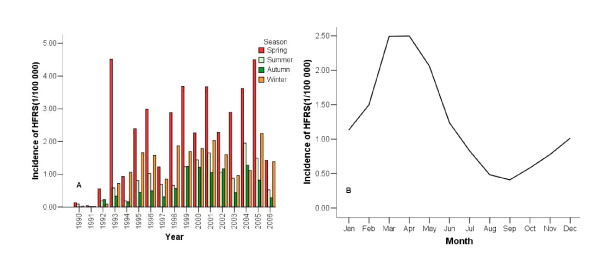
**Quarterly and monthly incidence of hemorrhagic fever with renal syndrome (HFRS) (Huludao City, China, 1990–2006)**. A) Quarterly incidence. B) Average monthly incidence of HFRS.

The HFRS incidence increased with a 2 to 3 years cycle from 1990 to 2006 in Huludao (Figure 2). There was a clear seasonal variation in the incidence of HFRS in Huludao City, with most cases acquired in the winter to spring, starting generally from December, reaching a peak in March/April and ending in June (Figure 3–A,B).

### Pearson's correlation analysis

Table 1 shows that temperature, precipitation, relative humidity and virus-carrying index were positively related to the monthly HFRS incidence with a lag of 3, 3, 3 and 1 month, respectively, while air pressure had a negative correlation with the incidence with a lag of 3 months.

**Table 1:** Relationship between major meteorological & reservoir factors and HFRS incidence (Huludao City, China, 1990–2006)

Meteorological & reservoir factors	Pearson correlation coefficients	Lag values(month)
Air pressure	-0.413(**)	3
Temperature	0.380(**)	3
Precipitation	0.270(**)	3
Relative humidity	0.380(**)	3
Virus-carrying index	0.497(**)	1

Note: ** Correlation is significant at the 0.01 level (2-tailed).

Lag1: one month prior

### Structure equation models

**Figure 4:**
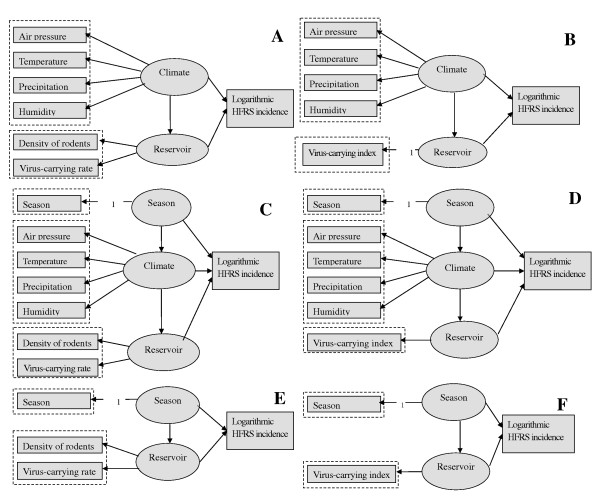
**Structure of the initial model–source of a prior hypothesis**.

Structure of the initial models is presented in Figure 4, two latent variables ("Climate" and "Reservoir") were adopted to explain the relationship between major meteorological and reservoir. Here, reservoir may indicate density of rodents and virus-carrying rate among rodents(Figure 4A) or only indicate one variable(Figure 4B), that is virus-carrying index among rodents. For the latter model, we let the regression coefficient between reservoir and virus-carrying index be 1.00. Because the influence of reservoir on climate was not empirically supported, we deleted the arrow from reservoir to climate from the initial model. Based on the results of Pearson's correlation analysis and HFRS's incubation period, lag period was set 3 months for climatic variables and one month for virus-carrying index. Due to the fact that in the study area the incidence of HFRS is clearly seasonal and climatic variables are clearly dependent on the season, we also fitted models where season was an independent variable. Season's possible combination with climate and reservoir was presented in Figure 4C, D, E and 4F.

**Figure 5:**
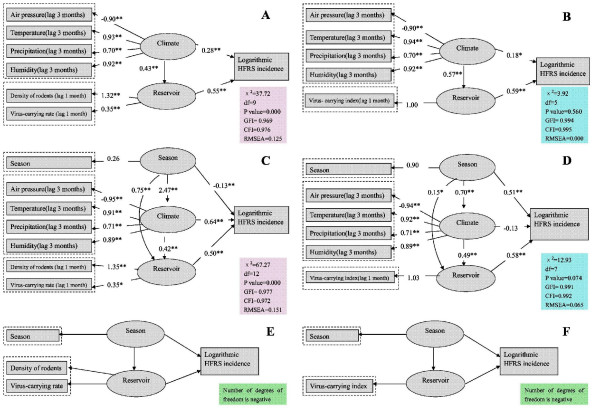
**Significant pathways of the final SEMs and goodness of fit indices (Huludao City, China, 1990–2006)**. Note: * Parameter is significant at the 0.05 level (2-tailed). ** Parameter is significant at the 0.01 level (2-tailed).

Among the constructed models, the last two models(Figure 5E and 5F) could not be established, degrees of freedom for both the models are negative. Goodness of fit indices indicated that the first(Figure 5A) and the third model(Figure 5C) should be rejected. The standardized regression coefficient between climate and HFRS incidence is positive in the second model(Figure 5B), while it is negative in the fourth model(Figure 5D). From the point of goodness of fit indices, the second model(Figure 5B) was accepted as the best-fit model. It produced a non-significant chi-square value, χ^2^(5) = 3.924, P value = 0.560 with GFI = 0.994, CFI = 0.995, NFI = 0.990, SRMR= 0.050, RMSEA = 0.000. This model gave an explained variance of 66% for HFRS incidence.

Figure 5B shows that climate and reservoir were positively correlated with the incidence of HFRS. Total effect of climate on HFRS incidence was 0.516 with a direct effect 0.18, and an indirect effect (through reservoir) 0.336 by multiplying 0.57 and 0.59, which means that climate affects HFRS incidence mainly through the effect on reservoir in the study area. As for each indicator, air temperature, precipitation and humidity 3 months prior were positively correlated with the incidence of HFRS and virus-carrying index one month prior. Air pressure 3 months prior was negatively correlated with HFRS incidence and virus-carrying index one month prior. The standardized regression coefficients are presented in Table 2 and 3.

**Table 2:** Relationship between the climatic variables and virus-carrying index in Huludao City, China, 1990 to 2006

Variables	Standardized Regression coefficients
Air pressure (lag 3 months)	-0.513 = -0.90 × 0.57
Air Temperature (lag 3 months)	0.536 = 0.94 × 0.57
Precipitation (lag 3 months)	0.399 = 0.70 × 0.57
Humidity (lag 3 months)	0.524 = 0.92 × 0.57

**Table 3:** Relationship of the measured indicators, latent variables and HFRS incidence in Huludao City, China, 1990 to 2006

Variables	Standardized Regression coefficients
Climate	0.516 = 0.18+0.57 × 0.59
Air pressure (lag 3 months)	-0.465 = -0.90 × 0.516
Air Temperature (lag 3 months)	0.485 = 0.94 × 0.516
Precipitation (lag 3 months)	0.361 = 0.70 × 0.516
Humidity (lag 3 months)	0.475 = 0.92 × 0.516
Virus-carrying index (lag 1 month)	0.59

## Discussion

The HFRS incidence increased from 1990 to 2006 in the study area. The reasons for the increase of HFRS incidence may be as follows: Firstly, more and more urban constructions were built and rebuilt in recent years. Rapid development of China's urban construction resulted in the frequent migration of *R. norvegicus *and more opportunities on contact with human being. Secondly, the increase of rural migrant workers resulted in the increase of susceptible population. Thirdly, the fundamental reason was that the density of rodents went upward with a high virus-carrying rate recently.

Epidemics of HFRS can fall with a well-defined periodicity and/or seasonality[38]. This variation is important because it can have significant implications for the design and effectiveness of control strategies. There is a clear seasonal variation of HFRS incidence in Huludao City and the incidence increased with a 2 to 3 years cycle during the study period. In China, at the national level, there was a peak of HFRS incidence every 8 years, at the county or city level, the cycle were 3 to 5 years[1]. The possible reason for the 2–3 year cycle in Huludao may be that the density of rodents and virus-carrying rate among rodents were at a high level during the study period. The density of rodents ranged from 1% to 12% with an average 5%, and virus-carrying rate among rodents ranged from 4% to 14% with an average 9%[33].

In 2005, the World Health Organization report on climate and prediction of infectious disease epidemics[39] indicated that the construction of weather- and climate-based systems to provide early warning of incipient epidemics was now feasible and could provide considerable population health benefit if informational, structural, and monetary barriers to implementation could be overcome. Several studies indicated the effect of climatic or reservoir variables on the incidence of HFRS, but few studies described the effect in quantity. Liaoning Province can be divided into three parts in respect to terrain: the Liaodong mountainous region, the Liaohe plain region, and the Liaoxi mountainous region[40]. Huludao City lies in the Liaoxi mountainous region. Lin H et al indicated that the terrain, the amount of forestation and the relative high humidity might be the important factors responsible for the epidemic development of HFRS in Liaoning Province, but they didn't explain what the association really was[41]. Our findings about the relationship between HFRS incidence, climatic factors and reservoir are of part concordance with the results of other studies in China[16,42,43]. Liu et al applied case-crossover design and conditional logistic regression to analyze the relationship between meteorological factors and HFRS incidence in one national surveillance spot of HFRS in Shandong province[42]. They found that mean temperature, rainfall, humidity sunlight, air pressure and velocity of wind were associated with HFRS incidence. Based on the surveillance data in Jiangsu Province, Wu et al indicated that meteorological factors could be used to predict HFRS incidence[43]. However, the climatic variables' effects on HFRS incidence via rodents were not considered in these studies.

In the present study, we discussed how climatic factors acted on reservoir and influenced the incidence of HFRS in the end. Because the climatic variables are not independent variables, the regression coefficients are very unstable with excessive standard error. We presented an SEM implementation to solve this problem. The final SEM indicated that climate affected HFRS incidence mainly through the effect on reservoir in the study area, reservoir had a greater effect in the model. The goodness of fit statistics meant that SEM was suitable for understanding such relational data in multivariate systems. The latent variable "climate" here refers to the average state over a longer time period[44]. SEM that had climatic variable with lagged HFRS incidence was designed because it allowed the examination of a lagged effect of climate variability to impact on the incidence of HFRS. The lag would capture the period of rodents growth, virus development time within the rodents and the virus incubation period within the human body[16]. The SEMs approach also has several limitations, such as 1) the idea that sample covariance instead of the sample values themselves was used to fit by modeling may destroy valuable data; 2) as the number of explanatory increases, the number of parameters in the model can increase exponentially; 3) SEM is usually viewed as a confirmatory rather than exploratory procedure, can't advance new casual model by itself.

As a national HFRS surveillance site, good records about HFRS incidence have been kept in Huludao City. The blood samples of HFRS cases were collected in the hospitals, serologic identification was then performed at the laboratory to confirm the clinical diagnosis. There might be admission rate bias in the disease report, but this has been reduced as much as possible[41]. The Chinese Government established a routine reporting system for selected infectious diseases in the 1950s, and the system switched from paper-based reporting to the submission of electronic files in 1985, and since 2003 has used web-based reporting[45]. Under-reporting is possible in the disease surveillance system, especially before the computer and web were widely applied. An investigation of missing reports of notifiable diseases in China in 2005 indicated that there was much to be improved upon in Chinese medical facilities as far as reporting of infectious diseases[46]. So, the relationship between climatic variables, reservoir information and HFRS may be underestimated. Further, in the present study only the new symptomatic HFRS cases were used to calculate the HFRS incidence, while, the majority of human hantavirus infections are asymptomatic[47]. In China, Song G reported that relatively high inapparent infection rates(8%–20%) in the population of endemic areas of the Rattus-type HFRS after big outbreaks played a significant role in the gradual decline of the incidence of HFRS [48], Bi P reported that the antibody tire of asymptomatic infection was too low to prevent the development of clinical cases[49]. The asymptomatic infection rate among healthy population in the study area varied from 3.6% to 9.2% with an average 5%, which may serve as a potential limitation of the study. Another limitation of the present study may be that we only focused on the relationship between the meteorological factors, reservoir factors and the incidence of HFRS. The occupational activities, such as farming and mining, may affect people's contact with rodents[16]. However, in epidemic foci of the Seoul virus, people who take different jobs have similar chance of contacting with *Rattus norvegicus *[1].

## Conclusion

In summary, SEM confirmed that climate and reservoirs have affected the incidence of HFRS in Huludao City, located in northeastern China. Climate affects HFRS incidence mainly through the effect on reservoir in the study area. HFRS prevention and control should give more consideration to rodent control and climate variations.

## Competing interests

The authors declare that they have no competing interests.

## Authors' contributions

PG conceived the study and drafted the manuscript. DH, PG and BZ secured funding. PG, DH and MH managed and analyzed the data. BZ contributed to the analysis design, and reviewed the manuscript. TS contributed to the HFRS incidence and reservoir data collection and managed the HFRS incidence database which was administered and supervised by JG. TS and MH contributed to the results interpretation. All authors contributed to the writing of the final version of this paper.

## Pre-publication history

The pre-publication history for this paper can be accessed here:

http://www.biomedcentral.com/1471-2334/9/109/prepub
